# Adiponectin is negatively associated with disease activity and Sharp score in treatment-naïve Han Chinese rheumatoid arthritis patients

**DOI:** 10.1038/s41598-022-06115-9

**Published:** 2022-02-08

**Authors:** Xixi Chen, Kaiwen Wang, Tao Lu, Jiajia Wang, Ting Zhou, Juan Tian, Bin Zhou, Li Long, Qiao Zhou

**Affiliations:** 1 Department of Rheumatology and Immunology, Sichuan Provincial People’s Hospital, University of Electronic Science and Technology of China, Chengdu, China; 2 Chinese Academy of Sciences Sichuan Translational Medicine Research Hospital, Chengdu, China; 3 School of Medicine, The University of Leeds, Leeds, UK; 4 Department of Radiology, Sichuan Provincial People’s Hospital, University of Electronic Science and Technology of China, Chengdu, China; 5 Department of Laboratory Medicine, Sichuan Provincial People’s Hospital, University of Electronic Science and Technology of China, Chengdu, China; 6 Department of Geriatrics, Sichuan Provincial People’s Hospital, University of Electronic Science and Technology of China, Chengdu, China; 7 The First People’s Hospital of Tianmen City, Tianmen, Hubei China

**Keywords:** Immunology, Biomarkers, Rheumatology

## Abstract

The association and potential role of the protein hormone adiponectin in autoimmune diseases causing musculoskeletal disorders, including rheumatoid arthritis (RA), are controversial. Conflicting results may arise from the influences of confounding factors linked to genetic backgrounds, disease stage, disease-modifying anti-rheumatic drugs and patients’ metabolic characteristics. Here, we examined serum level of adiponectin and its relationship with disease activity score 28 with erythrocytes sedimentation rate (DAS28[ESR]) and Sharp score in a treatment-naïve Han Chinese RA population. This cross-sectional study enrolled 125 RA patients. Serum level of total adiponectin was assessed by enzyme-linked immunosorbent assay (ELISA). Other important clinical and laboratory parameters were collected from the hospital database. DAS28(ESR) was calculated according to the equation previously published. Sharp score was evaluated based on hands radiographs by an independent radiologist. The correlation between serum adiponectin level and DAS28(ESR) or the Sharp score was investigated by univariate and multivariable linear regression analyses, respectively. Multiple imputation by chained equations was used to account for missing data. Univariate analyses showed a significant positive correlation between DAS28(ESR) and age or C-reactive protein (CRP) (both p = 0.003), while serum adiponectin level was negatively correlated with DAS28(ESR) (p = 0.015). The negative correlation between adiponectin level and DAS28(ESR) remained true in multivariable analyses adjusted for confounders. In addition, the univariate analyses revealed positive correlations of Sharp score to disease duration (p < 0.001), CRP (p = 0.023) and ESR (p < 0.001). In the multivariable model adjusted for confounders, adiponectin was negatively correlated with Sharp score (p = 0.013). In this single-institution cross-sectional study, serum adiponectin level in treatment-naive RA patients is negatively correlated with DAS28(ESR) and the Sharp score after adjustment for prominent identified confounders. Serum adiponectin may be potentially useful for assessing disease activity and radiographic progression of RA.

## Introduction

Rheumatoid arthritis (RA) is a chronic inflammatory-related autoimmune disease that primarily affects joints and causes musculoskeletal disorders^1^. The characteristics of RA include synovium hyperplasia, lymphocyte infiltration and abnormal proliferation of fibroblast-like synoviocytes, all of which leading eventually to erosive joint destruction^2^.

Adiponectin is a hormone protein mainly secreted by adipose tissue but also by various other cells, including skeletal myocytes and cardiomyocytes^3^. It is abundantly present in the circulation, accounting for 0.01% of total plasma proteins^4^. The potential role of adiponectin in RA has been actively investigated. Conflicting data on its role in RA have been reported. Adiponectin could act as proinflammatory mediator as its serum level positively correlates with disease activity^5–9^ or radiographic progression^10^. However, this association has not been unanimously agreed upon, with opposite results being reported from other studies^11–14^. Given these heterogenous findings coupled with the fact that most of these studies were conducted in Caucasian patients or with relatively higher body mass index (BMI)^15–17^ and some studies did not adjust for confounders^10^, we conducted a cross-sectional study to evaluate the relationship between adiponectin and disease activity as well as radiographic outcomes in a cohort of treatment-naïve Chinese RA patients using both univariate and multivariable linear regression methods.

## Materials and methods

### Study population

Between 2012 and 2020, one hundred twenty-five patients with rheumatoid arthritis diagnosed according to the American College of Rheumatology 1987 criteria were included in this study. Patients with co-morbidities such as hypertension, diabetes, hyperlipidaemia, chronic inflammatory disease, autoimmune disease, or cancer were excluded. At the time of the study, none of the patients had received treatment against RA. All patients belonged to the Han ethnic group. In addition to the RA patients, 34 healthy participants were studied to evaluate the baseline level of total serum adiponectin in the general population. The study was conducted in accordance with the Declaration of Helsinki and was approved by the Ethical Committee of Sichuan Provincial People’s Hospital. All subjects signed informed consent.

### Clinical and laboratory measurements

Clinical information and laboratory data were obtained through detailed interviews, self-reported questionnaires, physical examination, and blood tests. The BMI was calculated as [body weight/height^2^] (kg/m^2^). Disease activity was measured using modified disease activity score 28 with erythrocyte sedimentation rate (DAS28[ESR])^18^.

All bloods were collected in the morning, after overnight fasting. Levels of CRP, ESR, rheumatoid factor (RF) and anti-cyclic citrullinated peptide (CCP) antibodies were measured by the Clinical Laboratory of Sichuan Provincial People’s Hospital. Serum concentrations of total adiponectin were measured by enzyme-linked immunosorbent assay (ELISA), using the kit purchased from BioVision, USA. Samples were prepared at appropriate dilutions and assayed according to the manufacturer’s protocol.

### Radiographic outcomes

Single-view anterior–posterior X-rays of both hands were scored using the van der Heijde modification of the Sharp method (SHS) (referred to as Sharp score) by a single experienced reader blinded to patient characteristics^19^.

### Statistical analysis

Continuous variables were expressed as means ± standard deviations, and categorical data were expressed as a number (percentage). Statistical significance was calculated using *t* test (normal distribution) or Kruskal–Wallis test (skewed distribution) unless stated otherwise. A univariate linear regression model was used to analyse each variable’s relationship with DAS28(ESR) or Sharp score where results were presented as regression coefficient β and 95% confidence interval (95% CI). Next, multivariable linear regression models were performed against DAS28(ESR) or Sharp score to examine the β and 95% CI of serum adiponectin level after adjusting for confounders. Gender was selected to adjust for possible differential relationship between genders. Other variables were selected as confounders if they were either significantly associated with the outcome, or if when added to the basic model or removed from the full model, a change in effect estimate of adiponectin level by more than 10% was observed. ‘Basic model’ refers to the unadjusted univariate linear regression model between adiponectin and the outcome, whereas ‘full model’ refers to multivariable linear regression model adjusting for all covariates. Swollen joint count (SJC), tender joint count (TJC) and ESR were excluded from the full model of DAS28(ESR) because they were already captured by the score.

Missing data were noticed in age, disease duration, height, weight, ESR, CRP, RF, CCP, SJC, TJC, Sharp score and adiponectin. To maximise statistical power and minimise potential bias, we used multiple imputation by chained equations to create five imputed datasets to account for missing data^20^. Analyses were repeated on each of the imputed datasets and final results were obtained by combining the results from each individual analysis according to Rubin’s rules^21^. In addition, sensitivity analysis was performed to identify whether the created complete data had a significant difference from pre-imputation data. Moreover, smooth curve fitting was used to further characterize the nature of the associations between adiponectin and DAS28(ESR) or Sharp score. All the analyses were performed with R software Version 3.4.3 (http://www.R-project.org) and EmpowerStats (http://www.empowerstats.com, X&Y Solutions, Inc., Boston, MA). A P value less than 0.05 was considered statistically significant.

### Ethics approval and consent to participate

This study was approved by the Ethical Committee of Sichuan Provincial People’s Hospital. All subjects signed informed consent.

### Consent for publication

All patients signed a consent for publication form.

## Results

### Clinical and laboratory characteristics of the patients

The characteristics of the healthy controls are summarised in Supplementary Table S1 (Additional file 1). The clinical and laboratory profiles of the RA patients are summarised in Table 1. A total of 125 RA patients with a mean age of 55.7 ± 12.4 years were included, of which 94 (75.2%) were female. Compared to male patients, female patients were significantly younger (54.4 ± 12.7 vs 59.9 ± 10.1, p = 0.033). The average height and weight also showed significant differences between genders (both with p < 0.001), although the two groups had a compatible average BMI, which was overall of 22.6 ± 3.7. The mean DAS28(ESR) of the whole cohort was 5.4 ± 3.3 and the mean value of serum total adiponectin was 25.0 ± 19.1 μg/mL. Serum total adiponectin in an age- and sex-matched healthy population sample was significantly lower (13.6 ± 5.5 μg/mL; p = 0.015) compared with the RA group.

**Table 1:** Clinical and laboratory characteristics of the enrolled patients.

Characteristic	N (%)	Male	Female	Total	p value
Gender (N, %)	125 (100)	31 (24.8)	94 (75.2)		
Age (year)	124 (99.2)	60.9 ± 11.4	54.0 ± 12.3	55.7 ± 12.4	**0.007**
Height (cm)	122 (97.6)	167.2 ± 8.2	155.5 ± 5.0	158.4 ± 7.8	**< 0.001**
Weight (kg)	122 (97.6)	64.3 ± 11.0	54.4 ± 10.3	56.8 ± 11.3	**< 0.001**
BMI (kg/m^2^)	122 (97.6)	23.0 ± 3.2	22.4 ± 3.8	22.6 ± 3.7	0.504
Disease duration (month)	118 (94.4)	105.2 ± 116.4	109.0 ± 107.3	108.0 ± 109.1	0.871
SJC	123 (98.4)	4.5 ± 6.0	3.8 ± 6.3	4.0 ± 6.2	0.637
TJC	123 (98.4)	6.8 ± 8.7	6.9 ± 9.0	6.9 ± 8.9	0.844
CRP (mg/dL)	91 (72.8)	33.9 ± 38.3	40.1 ± 57.3	38.5 ± 52.8	0.627
ESR (mm/H)	124 (99.2)	52.8 ± 30.9	56.2 ± 33.0	55.4 ± 32.4	0.618
DAS28(ESR)	123 (98.4)	5.7 ± 3.4	5.3 ± 3.3	5.4 ± 3.3	0.594
RF (IU/mL)	122 (97.6)	444.2 ± 685.0	321.3 ± 469.3	352.5 ± 532.0	0.269
Anti-CCP (Ru/mL)	119 (95.2)	242.5 ± 173.1	230.8 ± 170.4	233.7 ± 170.4	0.746
Adiponectin (μg/mL)	118 (94.4)	21.4 ± 20.3	26.3 ± 18.6	25.0 ± 19.1	0.223
Sharp score	115 (92.0)	43.1 ± 63.5	44.3 ± 51.7	44.0 ± 54.7	0.917

*BMI* body mass index, *CCP* cyclic citrullinated peptides, *CRP* C-reactive protein, *DAS28* disease activity score of 28 joints, *ESR* erythrocyte sedimentation rate, *RF* rheumatoid factor, *SJC* swollen joint count, *TJC* tender joint count. Continuous variables are expressed as means ± standard deviations and categorical data using number (percentage). p < 0.05 is considered statistically significant and these values are in bold.

### Identification of factors correlated with DAS28(ESR) and Sharp score by univariate linear regression analysis

To ensure the correct measurement of adiponectin, we first investigated whether it was correlated with BMI. We found that adiponectin was negatively correlated with BMI (r = − 0.2571, p = 0.0055). The relationships between different clinical parameters and disease activity measured by DAS28(ESR) were then assessed by univariate linear regression analysis. Analyses using the univariate model against DAS28(ESR) indicated significant positive correlations with age and CRP (p = 0.0026 and p = 0.003, respectively; Table 2). In contrast, serum adiponectin was negatively correlated with DAS28(ESR) (p = 0.015). This model did not detect any further significant association between DAS28(ESR) and other variables (p > 0.05) (Table 2).

**Table 2:** Univariate linear regression results against DAS28(ESR) or Sharp score.

Variables	DAS28 (ESR)		Sharp score	
	β (95% CI)	p	β (95% CI)	p
**Gender**				
Male	0		0	
Female	− 0.27 (− 1.62, 1.09)	0.698	1.22 (− 21.65, 24.09)	0.917
Age (year)	0.07 (0.03, 0.12)	**0.003**	0.62 (− 0.17, 1.41)	0.128
Disease duration (month)	0.00 (− 0.00, 0.01)	0.177	0.30 (0.22, 0.38)	**< 0.001**
Height (cm)	− 0.03 (− 0.10, 0.05)	0.497	− 0.26 (− 1.54, 1.01)	0.688
Weight (kg)	− 0.03 (− 0.08, 0.03)	0.331	− 0.68 (− 1.58, 0.22)	0.141
BMI (kg/m^2^)	− 0.06 (− 0.23, 0.10)	0.444	− 2.23 (− 5.03, 0.56)	0.121
RF (IU/mL)	− 0.00 (− 0.00, 0.00)	0.886	0.01 (− 0.01, 0.03)	0.311
CCP (Ru/mL)	− 0.00 (− 0.01, 0.00)	0.204	− 0.01 (− 0.07, 0.03)	0.853
CRP (mg/dL)	0.02 (0.01, 0.03)	**0.003**	0.26 (0.04, 0.049)	**0.023**
ESR (mm/H)	N/A	N/A	0.65 (0.36, 0.94)	**< 0.001**
SJC	N/A	N/A	2.05 (0.51, 3.59)	**0.010**
TJC	N/A	N/A	2.07 (1.02, 3.13)	**< 0.001**
Adiponectin (μg/mL)	− 0.04 (− 0.07, − 0.01)	**0.015**	− 0.39 (− 0.99, 0.20)	0.198

*BMI* body mass index, *CCP* cyclic citrullinated peptides, *CRP* C-reactive protein, *RF* rheumatoid factor, *SJC* swollen joint count, *TJC* tender joint count. p < 0.05 is considered statistically significant and these values are in bold.

Analyses using the univariate model against the Sharp score revealed positive correlations with disease duration, CRP, ESR, SJC and TJC (p < 0.001, p = 0.023, p < 0.001, p = 0.010 and p < 0.001, respectively), whereas no correlation was observed with serum adiponectin level (Table 2).

### Analyses by multivariable linear regression showed an independent relationship between adiponectin level and DAS28(ESR) or Sharp score

We further explored the relationship between adiponectin level and the outcomes using multivariable linear regression analyses adjusted for confounders. Age, BMI, gender and CRP were finally selected as confounders for DAS28(ESR). Age, BMI, gender, disease duration, CRP, ESR, SJC and TJC were finally selected as confounders for Sharp score.

**Figure 1:**
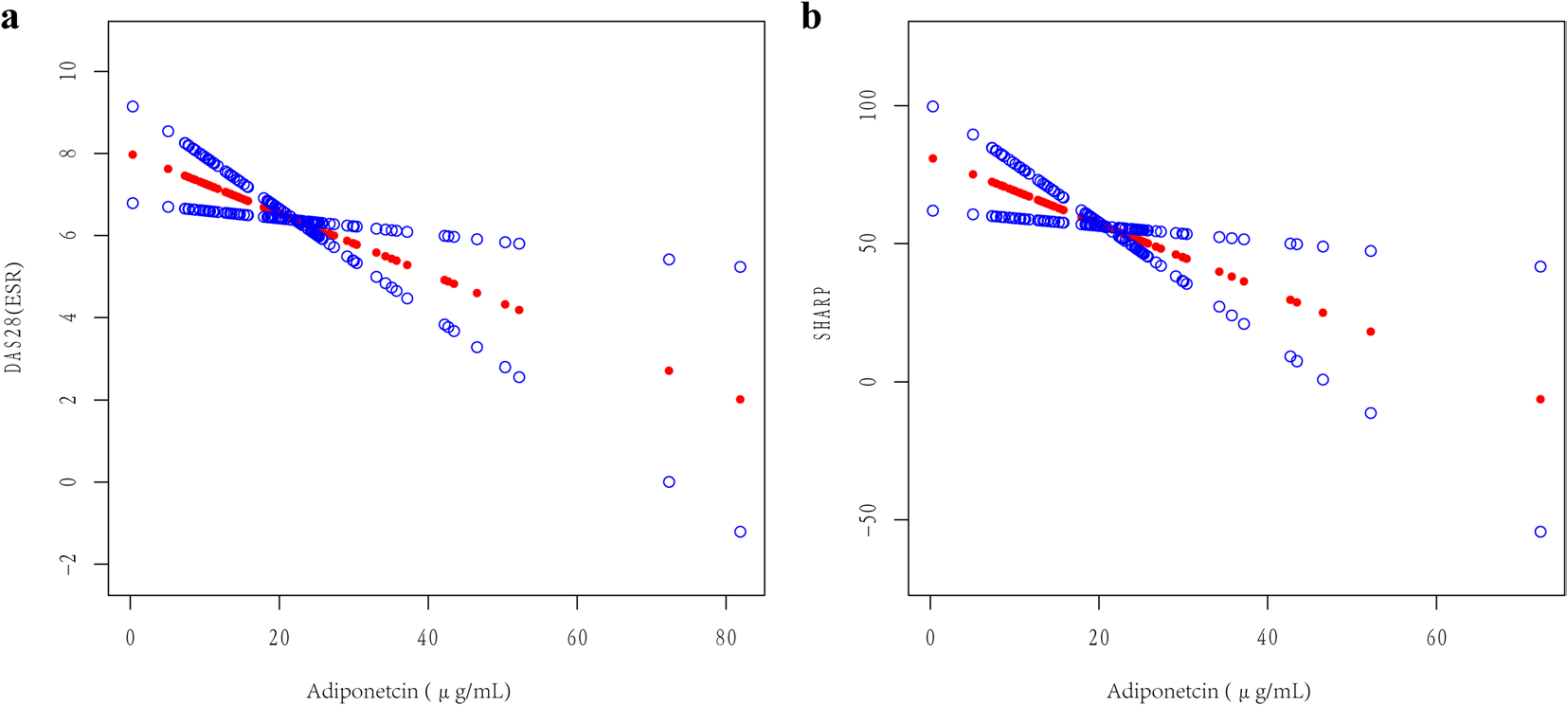
Association between serum adiponectin level and DAS28(ESR) (**a**) or Sharp score (**b**). The smooth curve fitting presented linear association between serum adiponectin and the two outcome measures. The red dotted line and blue dotted line represent the estimated values and their corresponding 95% confidence intervals (CI). Adjusted variables include age, BMI, gender and CRP for DAS28(ESR) and age, BMI, gender, disease duration, CRP, ESR, SJC and TJC for Sharp score.

In the crude model, there was no adjustment for confounders, while in model I, three confounders were adjusted when using DAS28(ESR) and four confounders were adjusted when using the Sharp score. Model II was adjusted for all confounders. DAS28(ESR) and adiponectin were significantly and negatively correlated in all three models (Table 3). In the models against the Sharp score, after adjusting for confounders, there was also a negative association between adiponectin level and Sharp score (Table 3). Further analysis using smooth curve fitting confirmed that the associations of adiponectin with DAS28(ESR) or Sharp score was linear after adjusting for all confounders (Fig. 1).

**Table 3:** Multivariable linear regression results against DAS28(ESR) or Sharp score, β (95% CI) of adiponectin (μg/mL).

Outcomes	Crude model	p	Model I	p	Model II	p
DAS28(ESR)	− 0.04 (− 0.07, − 0.01)	0.015	− 0.05 (− 0.08, − 0.01)^a^	0.007	− 0.07 (− 0.13, − 0.02)^b^	0.008
Sharp score	− 0.39 (− 0.99, 0.20)	0.198	− 0.72 (− 1.23, − 0.20)^c^	0.008	− 1.21 (− 2.14, − 0.28)^d^	0.013

Crude model: univariate model.

^a^Adjusted for age, BMI and gender.

^b^Adjusted for age, BMI, gender and CRP.

^c^Adjusted for age, BMI, gender and disease duration.

^d^Adjusted for age, BMI, gender, disease duration, CRP, ESR, SJC and TJC.

p < 0.05 is considered statistically significant.

### Sensitivity analysis

The amount of missing data for the different variables ranged from 0 to 27% (Table 1). Eighty-four out of the 125 (67%) patients had complete data for all variables for the main analyses. The distributions of the original and imputed variables are depicted in Supplementary Table S2 (Additional file 1). Regression analyses using multiple imputed datasets gave similar results to those undertaken on the original datasets, as displayed in Supplementary Table S3 (Additional file 1).

## Discussion

To evaluate the relationships between serum adiponectin and RA disease activity, we performed a cross-sectional investigation on a Chinese RA population. Adiponectin was negatively correlated with BMI in our study, which is in agreement with other studies^22,23^, validating the accuracy of our data. After accounting for confounders, multivariable linear regression analyses showed a negative correlation between serum adiponectin level and DAS28(ESR) or Sharp score. The fully-adjusted smooth curve fitting also confirmed a linear association between adiponectin with the outcomes. Since adiponectin was not normally distributed in our cohort, cube root transformation was performed on it and the conclusions remained the same (data not shown). Analyses were repeated after removing potential outlier noted in Fig. 1 (data not shown) and again all conclusions remained the same. To further avoid bias from missing data, we carried out a sensitivity analysis using full imputed data. With this sensitivity analysis, the associations of DAS28(ESR) and Sharp score with serum adiponectin remained statistically significant. These results are in keeping with previous studies showing that serum adiponectin level negatively correlated with disease activity in RA^15,24^.

In vitro experiments with RA synovial fibroblasts indicated that adiponectin significantly inhibits IL-1-induced RA synovial fibroblasts proliferation^25^. In a DBA/1 mouse model of collagen-induced arthritis, adiponectin treatment significantly mitigated the severity of arthritis along with a decrease in the expression of TNF-α, IL-1 and MMP-3 in joint tissues^26^. Anti-TNF treatment in female patients with RA was associated with increased adiponectin levels, which may dampen the systemic inflammatory response associated with RA^27^. These findings support an anti-inflammatory role for adiponectin in RA. However, several studies, including a metanalysis^16^, led to the conclusion that there was either no or a positive correlation between adiponectin level and inflammatory markers such as chemokines, CRP or DAS28^5,6,28,29^. This inconsistency may stem from the study designs and sampled populations, but also indicates that adiponectin may play different roles in inflammation, depending on disease characteristics, comorbidities, treatment, genetic backgrounds and physiological characteristics of the patients.

In addition to its role in inflammation, adiponectin has been shown to be protective against insulin resistance (IR) and diabetes^30^. High total adiponectin concentrations were significantly associated with lower risk for incident type 2 diabetes^30^. However, conflicting results on the association and prognostic value of blood adiponectin level in patients with coronary artery disease (CAD) have been reported. Some studies found that plasma adiponectin levels were significantly lower in patients with CAD than control subjects^31,32^, while a recent meta-analysis indicates that elevated adiponectin level is an independent predictor of cardiovascular and all-cause mortality in CAD patients^33^. It is known that RA patients have a 1.5–2.0 fold increased risk of developing CAD compared with the general population^34,35^, and the prevalence of IR is also higher in RA patients than in the general population (51–58% in RA vs 19% in controls)^36–38^. Therefore, combined analysis of inflammation markers and adiponectin could provide us a better understanding of both disease activity and metabolic status in RA patients. However, since patients with co-morbidities such as CAD, hypertension, diabetes, hyperlipidaemia were excluded from our study to avoid interfering with the research purposes, the role of adiponectin in metabolic disorder in our cohort is unclear and further analysis in real-world RA patients is warranted so as to explore the function of adiponectin in the setting of inflammation and metabolic disorder.

Another specificity of our study is that the enrolled patients had relatively long disease duration, high DAS28(ESR) and had not received any treatment for RA. The data from Chinese Registry of Rheumatoid Arthritis showed a mean 2.5-year delay in the diagnosis of RA after symptoms onset, 34.82% Chinese RA patients were found to have moderate disease activity and 47.18% to have high disease activity^39^. In our study, RA patients had an average disease duration of 108.0 ± 109.1 months without receiving adequate treatment and average DAS28(ESR) was 5.4 ± 3.3, which is classified as high disease activity. There are several challenges in the management of RA in our province which locates in southwest of China: (1) Public awareness is low and even some medical care professionals have limited knowledge of RA. (2) RA patients are unaware of the importance of standard treatment for better prognosis. Some believe in “folk medicine” and “radical therapy”. Until the disease progressed to late stage with severe deformity would they come to seek standard treatment. (3) Many primary care hospitals do not have department of rheumatology and therefore RA is often misdiagnosed. Disease duration has been demonstrated by previous studies as a significant independent predictor of joint damage, that is, bony erosions and joint space narrowing may not occur in the early stage of the disease^40^. In our study, 115/125 (92%) patients had an evaluable sharp score. Despite the fact we adjusted disease duration in the analysis of the association between adiponectin and Sharp score, whether our conclusion remains valid in patients with a shorter course still needs further verification, especially for patients with early arthritis, when there might be no radiographic change. Other methods, such as ultrasound or MRI, might be adopted to assess the relationship between adiponectin and the imaging outcome in patients within this period. On the other hand, different treatments may affect the level of adiponectin differentially^3,27,28,41,42^. Therefore, the absence of potential interferences between disease-modifying drugs and adiponectin level may have facilitated the demonstration of negative correlations between adiponectin level and DAS28(ESR) or Sharp score. Similarly, the relative homogeneity of our cohort regarding its ethnicity may have diminished the influence of potential confounding factors such as genetic background, BMI^43,44^ and adiponectin variants^45^ on the association between the adiponectin level and disease activity^7^.

Finally, some limitations and specificities should be taken into consideration when interpreting the data from our study. First, this is a cross-sectional clinical study without longitudinal data, and our results do not imply causation. Second, the participants were restricted to Chinese ethnicity from one district and predominantly females. It is known that Chinese populations have lower average BMI compared to other populations. Finally, as discussed above, there might be unconsidered covariates affecting RA severity or serum adiponectin such as genetic variants (i.e., RA risk HLA alleles and small nucleotide polymorphisms [SNPs] in the adiponectin gene) or adiponectin isoforms^7^. Further molecular investigation involving in vitro experiments and genetic comparisons in different populations are needed to untangle the pathophysiological role of adiponectin in RA progression.

In conclusion, multivariable linear regression analysis showed a negative and independent correlation between serum adiponectin and DAS28(ESR) or Sharp score after adjusting for important clinical confounders. Therefore, measurement of serum adiponectin may be potentially useful for assessing disease activity and radiographic damage of RA in Chinese patients regardless of ongoing medications.

## Supplementary Information

Supplementary Tables.

## Data Availability

The datasets used and/or analysed in the current study are available from the corresponding author upon reasonable request.

## Abbreviations

BMI: Body mass index
CCP: Cyclic citrullinated peptide
CRP: C-reactive protein
DAS28(ESR): Disease activity score 28 with erythrocyte sedimentation rate
ELISA: Enzyme-linked immunosorbent assay
ESR: Erythrocyte sedimentation rate
RA: Rheumatoid arthritis
RF: Rheumatoid factor
SJC: Swollen joint count
SNPs: Small nucleotide polymorphisms
TJC: Tender joint count
