# Supplementary material for: Adiponectin is negatively associated with disease activity and Sharp score in treatment-naïve Han Chinese rheumatoid arthritis patients
Source: Sci Rep. 2022 Feb 8;12:2092. doi: 10.1038/s41598-022-06115-9 (PMC8826401; doi:10.1038/s41598-022-06115-9)
Supplement: Supplementary file 1 — Supplementary Tables. [file 41598_2022_6115_MOESM1_ESM.docx]

Supplementary Appendix

Table of Contents

**Supplementary Table S1.** Clinical and laboratory characteristics of the enrolled healthy controls (pages 2).

**Supplementary Table S2.** Distribution of observed and imputed data (pages 3–4).

**Supplementary Table S3.** Multivariable linear relationship stratified by gender, β (95% CI) of adiponectin (μg/mL) — a sensitivity analysis with post-imputation data (page 5).

**Supplementary Table S1** Clinical and laboratory characteristics of the enrolled healthy controls

| Characteristic | Male | Female | Total | P value |
| --- | --- | --- | --- | --- |
| N (%) | 9 (26.4) | 25 (73.5) | 34 (100) |  |
| Age (years) | 59.1 ± 10.5 | 53.5 ± 9.3 | 55.1 ± 9.9 | 0.016 |
| Height (cm) | 170.7 ± 8.1 | 156.5 ± 5.3 | 159.8 ± 4.3 | < 0.001 |
| Weight (kg) | 67.3 ± 7.9 | 55.9 ± 8.6 | 58.9 ± 12.1 | < 0.001 |
| BMI (kg/m2) | 24.4 ± 1.3 | 22.9 ± 4.0 | 23.7 ± 2.9 | 0.099 |
| Adiponectin (μg/mL) | 12.5 ± 4.0 | 15.2 ± 6.2 | 13.6 ± 5.5 | 0.058 |

| **Supplementary Table S2.** Distribution of observed and imputed data. | | | | | | | |  |
| --- | --- | --- | --- | --- | --- | --- | --- | --- |
| Variables | Observed | Imputed 1 | Imputed 2 | Imputed 3 | | Imputed 4 | Imputed 5 | P value* |
| Female, n (%) | 94 (75.2) | 94 (75.2) | 94 (75.2) | 94 (75.2) | | 94 (75.2) | 94 (75.2) | 1.000 |
| Age (years) | 55.7 ± 12.4 | 55.7 ± 12.3 | 55.6 ± 12.3 | 55.5 ± 12.4 | | 55.5 ± 12.4 | 55.6 ± 12.3 | 0.999 |
| Height (cm) | 158.4 ± 7.8 | 158.3 ± 7.7 | 158.4 ± 7.8 | 158.4 ± 7.7 | | 158.6 ± 8.2 | 158.7 ± 8.3 | 0.991 |
| Weight (kg) | 56.8 ± 11.3 | 56.9 ± 11.2 | 57.0 ± 11.3 | 56.7 ± 11.2 | | 56.7 ± 11.3 | 57.3 ± 11.7 | 0.999 |
| BMI (kg/m2) | 22.6 ± 3.7 | 22.6 ± 3.7 | 22.6 ± 3.7 | 22.5 ± 3.6 | | 22.5 ± 3.7 | 22.6 ± 3.7 | 0.999 |
| Disease duration (month) | 108.0 ± 109.1 | 105.4 ± 107.6 | 105.3 ± 108.9 | 104.9 ± 107.4 | | 104.6 ± 107.6 | 107.1 ± 109.5 | 0.999 |
| SJC | 4.0 ± 6.2 | 4.0 ± 6.2 | 4.0 ± 6.2 | 4.0 ± 6.2 | | 4.0 ± 6.2 | 4.0 ± 6.2 | 1.000 |
| TJC | 6.9 ± 8.9 | 6.9 ± 8.9 | 6.9 ± 8.9 | 6.9 ± 8.9 | | 6.9 ± 8.9 | 6.9 ± 8.9 | 1.000 |
| CRP (mg/dL) | 38.5 ± 52.8 | 31.0 ± 47.9 | 31.5 ± 48.6 | 30.5 ± 47.8 | 32.7 ± 51.1 | | 31.7 ± 51.5 | 0.986 |
| ESR (mm/H) | 55.4 ± 32.4 | 55.4± 32.4 | 55.4 ± 32.4 | 55.4 ± 32.4 | | 55.4 ± 32.4 | 55.4 ± 32.4 | 1.000 |
| DAS28(ESR) | 5.4 ± 3.3 | 5.4 ± 3.3 | 5.4 ± 3.3 | 5.4 ± 3.3 | | 5.4 ± 3.3 | 5.5 ± 3.3 | 0.999 |
| RF (IU/mL) | 352.5 ± 532.0 | 366.3 ± 564.3 | 351.1 ± 527.0 | 346.4 ± 527.2 | | 350.2 ± 528.5 | 347.1 ± 526.8 | 0.999 |
| Anti-CCP (Ru/mL) | 233.7 ± 170.4 | 225.1 ± 170.9 | 231.7 ± 169.7 | 228.7 ± 169.2 | | 231.2 ± 179.5 | 232.0 ± 168.0 | 0.964 |
| Adiponectin (μg/mL) | 25.0 ± 19.1 | 25.6 ± 19.0 | 24.1 ± 18.9 | 25.0 ± 18.7 | | 24.6 ± 18.7 | 25.2 ± 20.3 | 0.991 |
| Sharp score | 44.0 ± 54.7 | 44.0 ± 54.7 | 44.0 ± 54.7 | 44.0 ± 54.7 | | 44.0 ± 54.7 | 44.0 ± 54.7 | 1.000 |

Abbreviations: BMI: body mass index; CCP: cyclic citrullinated peptides; CRP: C-reactive protein; DAS28: disease activity score of 28 joints; ESR: erythrocyte sedimentation rate; RF: rheumatoid factor; SJC: swollen joint count; TJC: tender joint count. Continuous variables are expressed as mean ± standard deviation and categorical data using number (percentage).

*Statistical significance was calculated using modified robust Brown-Forsythe Levene-type test based on the absolute deviation from the median.

**Supplementary Table S3.** Multivariable linear relationship stratified by gender, β (95% CI) of adiponectin (μg/mL) — a sensitivity analysis with post-imputation data

| Outcomes | Crude model | P | Model I | P | Model II | P |
| --- | --- | --- | --- | --- | --- | --- |
| DAS28(ESR) | -0.04 (-0.07, -0.00) | 0.028 | -0.04 (-0.08, -0.00)^a^ | 0.016 | -0.04 (-0.07, -0.00)^b^ | 0.027 |
| Sharp score | -0.55 (-1.12, 0.03) | 0.064 | -0.73 (-1.22, -0.23)^c^ | 0.004 | -0.58 (-1.06, -0.10)^d^ | 0.019 |

Crude model: Univariate model

a: adjusted for age and BMI and gender.

b: adjusted for age, BMI, gender and CRP.

c: adjusted for age, BMI, gender and disease duration.

d: adjusted for age, BMI, gender, disease duration, CRP, ESR, SJC and TJC.
